# One Patient: Two Variants of Takotsubo Cardiomyopathy

**DOI:** 10.7759/cureus.49203

**Published:** 2023-11-21

**Authors:** Mohamed Ramzi Almajed, Ahmed Babwi, Mustafa Mohammed, Sarah Gorgis, Zain Azzo, Sachin Parikh

**Affiliations:** 1 Internal Medicine, Henry Ford Hospital, Detroit, USA; 2 Cardiology, Henry Ford Hospital, Detroit, USA

**Keywords:** stress-induced cardiomyopathy, takotsubo cardiomyopathy, stress-related cardiomyopathy, double takotsubo, reverse takotsubo, atypical takotsubo, typical takotsubo

## Abstract

Takotsubo cardiomyopathy (TCM) is a form of non-ischemic cardiomyopathy that can present with signs of heart failure and volume overload; it often mimics acute coronary syndrome. It is characterized by stress-induced transient left ventricular (LV) dysfunction. Echocardiography classically demonstrates LV apical ballooning and akinesis in typical TCM, although other less common variants exist. Patients typically present with one variant.

A 32-year-old woman with a past medical history of alcohol use disorder, anxiety, and hypertension presented to the hospital with chest pain, shortness of breath, nausea, vomiting, and diarrhea. She was diagnosed with cardiogenic shock in the setting of a newly identified LV ejection fraction (EF) of 24% on echocardiogram with findings consistent with typical apical TCM. Ischemic workup was unremarkable, and she was medically managed with clinical improvement and subsequent recovery of cardiac function. Four months later, the patient presented with similar symptoms at which time she was found to have a recurrence of heart failure with reduced LV EF; echocardiography showed reverse TCM.

Patients with TCM who develop a recurrence typically maintain the same variant. The recurrence of TCM in a single patient with different anatomical variants is rare and poorly understood. We presented a case of a patient with alcohol use disorder who developed a recurrence of TCM with two anatomical variants. Further studies are necessary to investigate the predictors of recurrence and better understand the underlying mechanisms behind the different variants.

## Introduction

Takotsubo cardiomyopathy (TCM), or stress-induced cardiomyopathy, is a syndrome of cardiac dysfunction that was first described in 1990 by Sato et al. [1]. It is characterized by transient regional left ventricular (LV) dysfunction with a severe reduction of ejection fraction (EF) in the absence of significant coronary artery disease. Clinical presentation is variable and most commonly involves chest pain or dyspnea [2-3]. Investigations often show electrocardiograms with features concerning myocardial ischemia and elevated cardiac biomarkers. Echocardiography shows a reduced LV EF with abnormal wall movement [4].

Women are disproportionately affected as 90% of identified cases occur in post-menopausal women between the ages of 58 and 75 [5]. The pathophysiology of TCM involves a stress-induced catecholamine surge, which causes myocardial dysfunction; physical and emotional stress are, therefore, inciting factors. Alternative causes of myocardial dysfunction including ischemia and myocarditis are ruled out to attribute the presentation to non-ischemic cardiomyopathy [6].

TCM anatomical variants have been extensively described based on the LV site involved in echocardiography. Four distinct morphologies are reported by the International Takotsubo Registry, which includes apical or typical, midventricular, basal or reverse, and focal [7]. The pattern of myocardial dysfunction involves a concentrated anatomic area that is not consistent with a single coronary artery territory.

Recurrence of TCM is rare but remains higher than the incidence of TCM in the general population, which is estimated to be less than 1% [8]. The incidence of recurrence has been reported in multiple studies and is variable; the largest multi-center study reports a 4% lifetime recurrence [9-12]. The time between the index episode and subsequent recurrence is variable. Risk factors for recurrence have not been clearly identified, although patients who experienced recurrence tended to be younger [9]. Patients with TCM who develop recurrence of the condition typically maintain the same variant [10]. The recurrence of TCM in a single patient with different anatomical variants is rare and poorly understood [11-12].

We present a case of a young woman with alcohol use disorder who experienced recurrent TCM with two distinct anatomical variants.

## Case presentation

A 32-year-old woman presented with three days of chest pain, shortness of breath, nausea, vomiting, and diarrhea. She had a past medical history of primary hypertension, anxiety, and moderate alcohol use disorder. Physical exam was notable for bilateral lower limb swelling and tremors concerning alcohol withdrawal.

**Figure 1:**
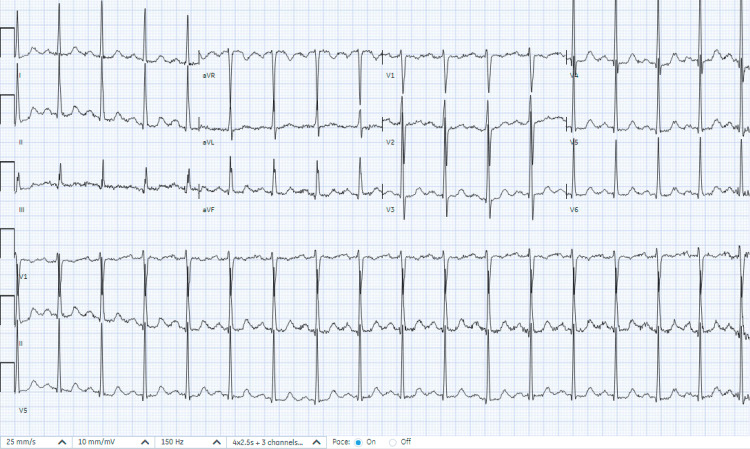
Electrocardiogram showing sinus tachycardia with non-specific T-wave changes

**Figure 2:**
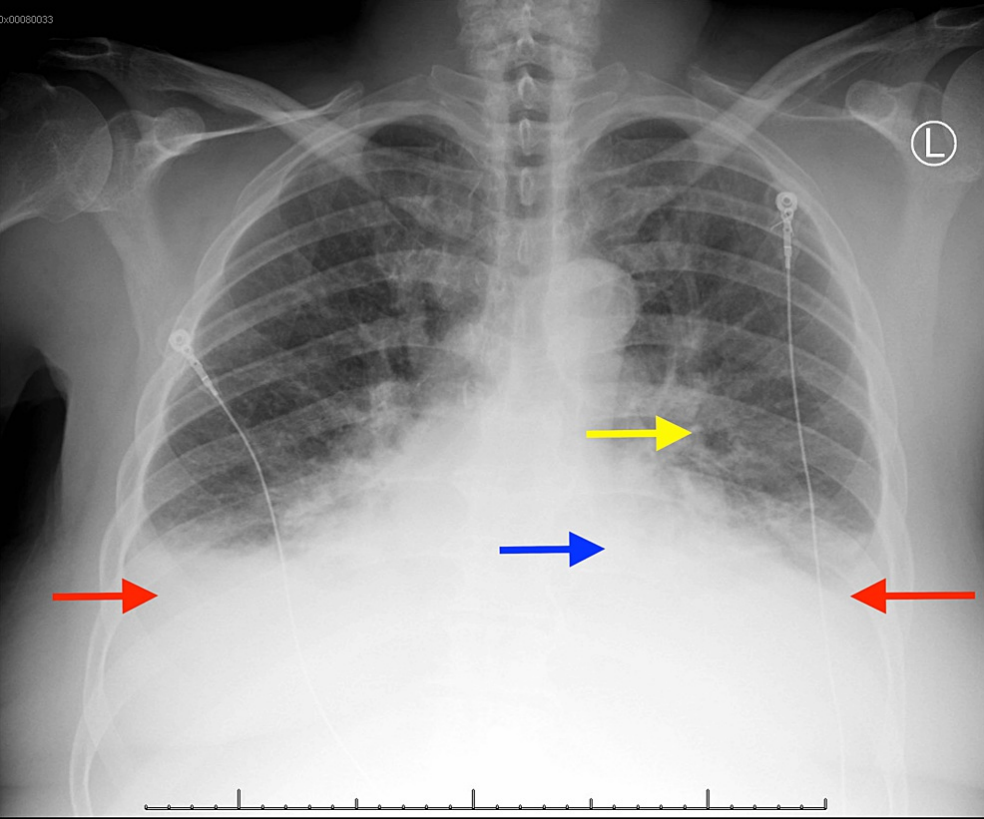
Chest x-ray showing bilateral pleural effusions and pulmonary vascular congestion suggestive of pulmonary edema Chest radiograph annotated demonstrating bilateral pleural effusions (red arrows), cardiomegaly (blue arrow), and pulmonary vascular congestion (yellow arrow)

Initial vital signs revealed tachycardia but were otherwise within normal limits. Laboratory investigations were significant for a lactate of 5.5 mmol/L (reference: <2.1 mmol/L) and an anion gap of 19, consistent with alcoholic ketoacidosis (Table 1). She had a high-sensitivity troponin I elevation with a peak of 10,995 ng/L (reference: <19 ng/L) and brain natriuretic peptide (BNP) of 970 pg/mL (reference: <50 pg/mL). An electrocardiogram showed sinus tachycardia with non-specific T-wave changes (Figure 1). Chest x-ray showed pulmonary vascular congestion with diffuse bilateral opacities concerning pulmonary edema (Figure 2). Limited bedside transthoracic echocardiogram noted a newly identified LV EF of 15%.

**Table 1:** Laboratory investigations BNP, brain natriuretic peptide

Laboratory Test (units)	Patient’s Result	Reference Range
Lactate (mmol/L)	5.5	<2.1
Anion Gap (N/A)	19	3-13
High-sensitivity Troponin I (ng/L)	10,995	<19
BNP (pg/mL)	970	<50

**Figure 3:**
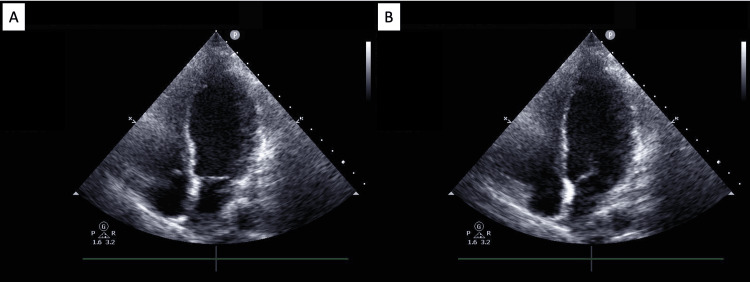
Transthoracic echocardiogram in apical four-chamber view demonstrating the patient’s first episode of TCM with apical variant morphology Apical TCM visualized in systole (1A) and diastole (1B) TCM, Takotsubo cardiomyopathy

**Figure 4:**
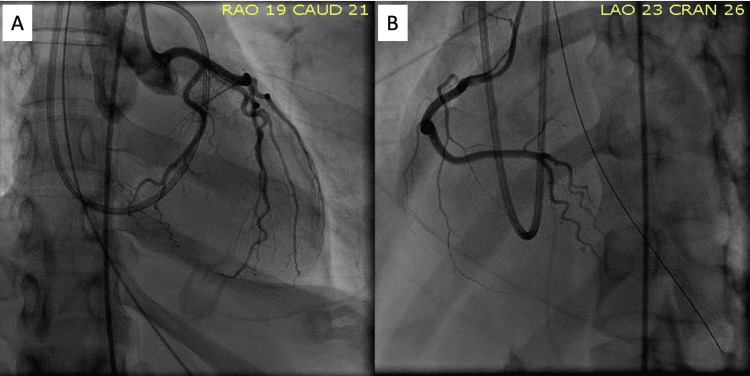
Coronary angiography showing the absence of obstructive coronary artery disease Absence of obstructive coronary artery disease visualized on coronary angiography in the RAO caudal view (2A) and LAO cranial view (2B) LAO, left anterior oblique; RAO, right anterior oblique

The patient was initially admitted to the general medical unit where she was managed for presumed acute coronary syndrome, heart failure with reduced EF, and alcohol withdrawal. Her course was complicated by hemodynamic instability and acute hypoxia requiring intubation after which she was transferred to the cardiovascular intensive care unit. A complete transthoracic echocardiogram was notable for an LV EF of 24% with severe global hypokinesis of the majority of the LV wall with relatively preserved function of the basal wall segments (Figure 3). Right and left heart catheterizations demonstrated non-obstructive coronary artery disease and a cardiac index of 1.22 L/min/m^2^ (Figure 4). She was treated for cardiogenic shock due to non-ischemic cardiomyopathy with milrinone and norepinephrine and was placed on mechanical circulatory support with an intra-aortic balloon.

Our patient had marked clinical recovery over the next three days, and she was weaned off hemodynamic support and extubated. A repeat echocardiogram three days later was notable for marked recovery in LV EF to 50% with mild hypokinesis of the LV basal inferior, anterior, mid-distal anteroseptal, and anterolateral walls. The diagnosis of apical variant TCM was made in the setting of these findings, and she was discharged on medical therapy for heart failure with a beta-blocker and angiotensin II receptor blocker (ARB). Repeat echocardiogram seven days and three months later showed normalization of cardiac function and a complete absence of LV wall motion abnormalities.

Four months after the patient’s initial presentation, she presented to the hospital with central chest pain that radiated to her left arm, diaphoresis, nausea, and tremulousness. She had stopped consuming alcohol for the past three days after having consumed six drinks of hard liquor daily the month prior. She had also been non-adherent to her medications.

**Figure 5:**
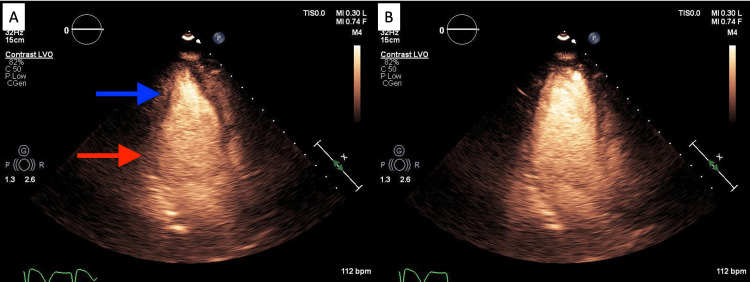
Transthoracic echocardiogram in apical four chamber view demonstrating the patient’s second episode of TCM with reverse variant morphology Reverse TCM visualized in systole (3A) and diastole (3B) with basal hypokinesis (red arrow) and apical normal contraction (blue arrow) TCM, Takotsubo cardiomyopathy

Vital signs revealed tachycardia and tachypnea but were otherwise within normal limits. Laboratory investigations were significant for a lactate of 4.5 mmol/L and an anion gap of 21. High-sensitivity troponin I and BNP were elevated at 8,894 ng/L and 111 pg/mL, respectively. The electrocardiogram showed non-specific T-wave changes. Transthoracic echocardiogram showed an LV EF of 38% with severe LV wall hypokinesis at the basal-to-mid LV wall segments in a noncoronary distribution; the apical wall segment was functioning normally (Figure 5). Heart catheterization was not performed given the low clinical suspicion of coronary obstruction and the unremarkable recent left heart catheterization.

The clinical picture was consistent with a recurrence of TCM with this episode manifesting with a different variant, reverse TCM. Our patient was medically managed for TCM and alcohol withdrawal after which she had clinical improvement and was subsequently discharged.

## Discussion

TCM is an uncommon diagnosis in patients thought to have acute coronary syndrome; it shares similar symptoms, electrocardiogram abnormalities including ST-segment changes, and cardiac biomarker elevation [6,13]. The prevalence of TCM is estimated at 5.2 cases per 100,000 women and 0.6 cases per 100,000 men in the United States [14]. Recurrence of TCM has been reported but remains rare given the prevalence rate; therefore, its risk factors and predictors remain unknown.

The pathogenesis of TCM has been hypothesized in many studies but the exact mechanism remains unclear. Patients who develop TCM are likely to have a genetic predisposition to an exaggerated myocardial response to stress. Stress-induced activation of biochemical and hormonal pathways is thought to be at the center of this syndrome. Inflammatory and catecholamine-driven myocardial damage occurs due to resultant catecholamine toxicity, microcirculatory dysfunction, transient ischemia from plaque rupture, and epicardial spasm [2-3,15]. In our patient, her two episodes of TCM occurred in the setting of alcohol withdrawal during which she was likely experiencing a profound catecholamine-mediated stress response. TCM has been reported in the setting of alcohol withdrawal across several cases in the literature [16-18].

The stress-induced cardiomyopathy in our patient was likely triggered by alcohol withdrawal on two separate occasions. It is important to note that alcohol use can induce cardiomyopathy, and patients often present with decompensated heart failure; however, if alcohol-associated cardiomyopathy is the sole reason for the cardiomyopathy, the clinical presentation and workup will be discordant to that of TCM. Chronic alcohol ingestion leads to an imbalance of neurotransmitters GABA-A and glutamate, with longer use resulting in decreased sensitivity of the receptors. The immediate cessation of alcohol use results in a hyperadrenergic state with a high level of plasma catecholamines in the first few days of detoxification and an increase in beta-adrenergic receptor sensitivity [19]. This physiologic stress and adrenergic surge can lead to myocardial stunning and transient cardiomyopathy.

Our patient interestingly experienced two episodes of TCM in the setting of alcohol withdrawal during which she had two different variants of the syndrome. There have only been a few reported cases of multiple TCM variants in one patient [9-11]. Most previously reported cases occurred in women aged 50 years and older. Previous studies have hypothesized that a relative decline in estrogen levels is linked to TCM recurrence in postmenopausal women [20]. Our patient was 32 years old at the time of her presentation, which suggests that other mechanisms could be contributing. Neurological and psychiatric disorders are consistent findings among these cases including our patient.

Age trends among patients with TCM consistently demonstrate a low prevalence among young patients, which further increases the rarity of our case in which a young patient presented twice with two variants of this condition. An analysis of the InterTAK Registry revealed that among all reported cases of TCM, only 10% involved patients aged 50 years and younger [21]. Among these younger patients, uncommon variants of TCM were disproportionately more common. Patients also had more severe presentations with a larger portion of them presenting with cardiogenic shock requiring advanced therapies for management including mechanical circulatory support. Moreover, this population had a higher prevalence of neurologic and psychiatric disorders, which supports prior observations and is consistent with our patient’s clinical vignette.

Based on the German Italian Spanish Takotsubo (GEIST) Registry, patients with recurrence had greater cardiovascular risk factors, but there was no significant difference in exposure to stressful events [9]. The earliest time for recurrence was within eight days of the index episode, and the latest recurrence was 2555 days afterward. Most cases of recurrent TCM were in the form of apical TCM, with a minority having a midventricular form; there were no cases of recurrent basal or focal variants. Among recurrent cases, 20% had a variant that was discordant with the initial presenting variant. No predictive factors for recurrence were found, and the mechanism for this remains unclear. It remains unknown why some patients are predisposed to a recurrence of TCM with different morphologies and the mechanism for this change.

Medical management of TCM has been associated with better outcomes and a decreased recurrence of TCM. The GEIST Registry noted a lower rate of recurrence in patients treated with a combination of a beta-blocker and an angiotensin-converting enzyme inhibitor (ACE-I) or ARB [9]. Another systematic review and meta-analysis of recurrent TCM found that ACE-I and ARB therapy was associated with a decreased recurrence of TCM, although beta-blocker therapy was not [22]. Moreover, patients with more severe initial presentations of TCM were more likely to develop recurrence. This finding is consistent with our case as our patient’s index presentation was that of a cardiogenic shock.

The clinical outcome of patients who develop TCM is favorable, as most patients experience a recovery in cardiac function, improvement in LV EF, and resolution of cardiac wall motion abnormalities [4,6,13]. Despite her severe presentation and recurrent episodes of TCM, our patient had a complete resolution in symptoms and recovery in cardiac function on subsequent follow-up. Best practice regarding monitoring and medical management following an episode of TCM is unclear and requires further research and expert consensus.

## Conclusions

Patients with TCM who develop a recurrence typically maintain the same variant. The recurrence of TCM in a single patient with different anatomical variants is rare and poorly understood. We presented a case of a patient with alcohol use disorder who developed a recurrence of TCM with two anatomical variants. This case highlights the need for further studies to investigate the predictors of recurrence and better understand the underlying mechanisms behind the different variants.
